# Supplementary material for: Upper critical solution temperature polymer assemblies via variable temperature liquid phase transmission electron microscopy and liquid resonant soft X-ray scattering
Source: Nat Commun. 2023 Jun 10;14:3441. doi: 10.1038/s41467-023-38781-2 (PMC10257671; doi:10.1038/s41467-023-38781-2)
Supplement: Supplementary file 1 — Supplementary Information [file 41467_2023_38781_MOESM1_ESM.pdf]

Upper Critical Solution Temperature Polymer Assemblies via Variable Temperature Liquid Phase Transmission Electron Microscopy and Liquid Resonant Soft X-Ray Scattering

Joanna Korpanty,[1] Cheng Wang,[2] and Nathan C. Gianneschi[1,3,4]

[1]Department of Chemistry, International Institute for Nanotechnology, Chemistry of Life Processes Institute, Simpson Querrey Institute, Northwestern University, Evanston, Illinois 60208, United States

[2]Advanced Light Source, Lawrence Berkeley National Laboratory, 1 Cyclotron Road, Berkeley, California 94720, United States

[3]Department of Materials Science & Engineering, Northwestern University, Evanston, IL, 60208, USA

[4]Department of Biomedical Engineering and Department of Pharmacology, Northwestern University, Evanston, IL, 60208, USA

*Corresponding author: nathan.gianneschi@northwestern.edu

Contents

I. COMSOL Radiolysis Modeling

II. Additional Data

III. Synthesis

IV. References

I. COMSOL Radiolysis Modeling

To create a model for solvent radiolysis, we utilized the reaction engineering module of COMSOL with the PARADISO solver. The concentration with time (*C(t)*) of each radiolytic product in each solvent was measured by summing the reaction rates of individual reactions (R_i) between radiolytic products and adding in the yield from direct irradiation (G_r), according to the equation:

$$C(t) = (\sum_i R_i) + G_r \quad (1)$$

Note that G_r is measured in units of M s^{-1} and is dependent on the G-value of the given radiolytic species (*G*, molecules/100 eV) and the dose rate (*D*, Gy s^{-1}), as shown in the equation below, where ρ is the density of the solvent (g cm s^{-3}) and *F* is Faraday's constant:[1]

$$G_r = \frac{\rho \times D \times G}{F} \quad (2)$$

For our modeling, we used a 300 keV electron beam with a 1 µm radius (*a*) and 1 nA current (*I*). The dose rate was calculated according to the equation below, where S is the density-normalized stopping power of the solvent:[1, 2]

$$D = \frac{S \times 10^5 \times I}{\Pi a^2} \quad (3)$$

Supplementary Table 1. G-values for isopropanol radiolysis.[3, 4, 5, 6, 7, 8, 9, 10, 11, 12, 13, 14, 15, 16, 17, 18, 19, 20, 21, 22, 23, 24, 25, 26, 27, 28, 29, 30, 31, 32, 33, 34, 35, 36, 37, 38, 39, 40, 41, 42, 43, 44, 45, 46, 47, 48, 49, 50, 51, 52, 53, 54, 55, 56, 57, 58]

Species	G-value (molecules/100eV)
e^-_s	1.00
ether	0.10
CH_4	1.36
H_2	4.00

CO	0.19
dimer	0.51
$(CH_3)_2CO$	1.47
Glycols	0.20
CH_3CHO	0.30
H•	2.20
$•CH_3$	1.50
$(CH_3)_2COH•$	7.00

Supplementary Table 2. Rate constants for reactions between radiolytic products of isopropanol.[3, 4, 5, 6, 7, 8, 9, 10, 11, 12, 13, 14, 15, 16, 17, 18, 19, 20, 21, 22, 23, 24, 25, 26, 27, 28, 29, 30, 31, 32, 33, 34, 35, 36, 37, 38, 39, 40, 41, 42, 43, 44, 45, 46, 47]

**Reaction**	***k***
$(CH_3)_2CHOH + •OH \rightarrow •CH_3CH_2HOH + H_2O$	1.98×10^8 M^{-1} s^{-1}
$(CH_3)_2CHOH + •OH \rightarrow (CH_3)_2COH• + H_2O$	3.30×10^9 M^{-1} s^{-1}
$(CH_3)_2CHOH + •H \rightarrow H_2 + (CH_3)_2COH•$	9.00×10^8 M^{-1} s^{-1}
$(CH_3)_2COH• + •OH \rightarrow (CH_3)_2CO + H_2O$	1.44×10^{11} s^{-1}
$(CH_3)_2COH• \rightarrow CH_3CHO + •CH_3$	1.30×10^5 M^{-1} s^{-1}
$(CH_3)_2CHOH_2^+ + e^-_s \rightarrow H• + (CH_3)_2CHOH$	1.00×10^{11} M^{-1} s^{-1}
$(CH_3)_2CHOH + e^-_s \rightarrow H• + (CH_3)_2CHO^-$	1.00×10^5 M^{-1} s^{-1}
$H• + (CH_3)_2CO \rightarrow CH_3CH_2CO• + H_2$	4.03×10^5 M^{-1} s^{-1}
$(CH_3)_2CO + HO_2• \rightarrow H_2O_2 + CH_3CH_2CO•$	4.80×10^5 M^{-1} s^{-1}
$(CH_3)_2CO + •CH_3 \rightarrow CH_4 + CH_3CH_2CO•$	33 M^{-1} s^{-1}
$(CH_3)_2CO + •OH \rightarrow CH_3CH_2CO• + H_2O$	1.36×10^8 M^{-1} s^{-1}
$e^-_s + e^-_s \rightarrow H•$	1.00×10^{11} M^{-1} s^{-1}
$(CH_3)_2CO + e^-_s \rightarrow (CH_3)_2CO•^-$	1.00×10^{10} M^{-1} s^{-1}
$(CH_3)_2COH• + O_2 \rightarrow (CH_3)_2COHOO•$	1.80×10^9 M^{-1} s^{-1}
$•CH_3 + •O \rightarrow CH_2O + H•$	9.00×10^{10} M^{-1} s^{-1}
$•CH_3 + •CH_3 \rightarrow CH_3CH_3$	7.83×10^{10} M^{-1} s^{-1}
$O_2 + •CH_3 \rightarrow CH_3OO•$	5.00×10^8 M^{-1} s^{-1}
$CO + H• \rightarrow CHO•$	4.90×10^7 M^{-1} s^{-1}
$CO + CH_3• \rightarrow CH_3CO•$	2.00×10^6 M^{-1} s^{-1}

$CO + e^{-}_{s} \rightarrow CO\bullet^{-}$	5.00×10^{8} M^{-1} s^{-1}
$(CH_3)_2COH\bullet + (CH_3)_2COH\bullet \rightarrow (CH_3)_2CHOH + (CH_3)_2CO$	7.00×10^{9} M^{-1} s^{-1}
$H\bullet + H\bullet \rightarrow H_2$	7.75×10^{9} M^{-1} s^{-1}
$(CH_3)_2COHOO\bullet \rightarrow H^{+} + (CH_3)_2CO + O_2\bullet^{-}$	6.65×10^{5} s^{-1}
$(CH_3)_2CHOH + \bullet CH_3 \rightarrow CH_4 + (CH_3)_2COH\bullet$	405 M^{-1} s^{-1}
$(CH_3)_2COH\bullet + (CH_3)_2COH\bullet \rightarrow ((CH_3)_2COH)_2$	7.80×10^{8} s^{-1}
$(CH_3)_2CHOH + \bullet CH_3 \rightarrow (CH_3)_2CHOCH_3 + \bullet H$	1.00×10^{4} M^{-1} s^{-1}
$CH_3CHOH\bullet + (CH_3)_2COH\bullet \rightarrow (CH_3)_2COH\text{-}CHOHCH_3$	7.80×10^{8} M^{-1} s^{-1}
$CH_3CHOH\bullet + CH_3CHOH\bullet \rightarrow (CH_3HOHC)_2$	7.80×10^{8} M^{-1} s^{-1}
$(CH_3)_2CHOH + \bullet CH_3 \rightarrow CH_4\ (CH_3)_2COH\bullet$	429 M^{-1} s^{-1}
$(CH_3)_2COH\bullet + CH_3CHOH\bullet \rightarrow (CH_3)_2CHOH + CH_3CHO$	4.81×10^{9} s^{-1}
$CH_3CHOH\bullet + H\bullet \rightarrow CH_3CH_2OH$	2.65×10^{10} s^{-1}
$CH_3CHOH\bullet + O\bullet \rightarrow CH_3CHO + \bullet OH$	9.03×10^{10} M^{-1} s^{-1}
$CH_3CHOH\bullet + H\bullet \rightarrow CH_3CHO + H_2$	1.79×10^{7} M^{-1} s^{-1}
$CH_3CHOH\bullet + H\bullet \rightarrow C_2H_4 + H_2O$	6.02×10^{8} M^{-1} s^{-1}
$CH_3CHOH\bullet + H\bullet \rightarrow CH_4 + CH_2O$	5.56×10^{5} M^{-1} s^{-1}
$(CH_3)_2CHOH + OH\bullet \rightarrow (CH_3)_2CHO + H_2O$	2.12×10^{8} M^{-1} s^{-1}
$CH_3CH_2CHOH\bullet + (CH_3)_2CHOH \rightarrow (CH_3)_2COH\bullet$	53.0 M^{-1} s^{-1}
$CH_3CH_2CHOH\bullet + CH_3CH_2CHOH\bullet \rightarrow (CH_3CH_2CHOH)_2$	1.00×10^{9} M^{-1} s^{-1}
$\bullet CH_3 + H\bullet \rightarrow CH_4$	2.11×10^{11} M^{-1} s^{-1}
$(CH_3)_2COH\bullet + CH_2O \rightarrow (CH_3)_2COHCH_2O\bullet$	6.00×10^{3} M^{-1} s^{-1}
$\bullet CH_3 + CH_2O \rightarrow CH_4 + \bullet CHO$	7.00×10^{9} M^{-1} s^{-1}
$\bullet CH_3 + CH_2O \rightarrow CH_3CH_2O\bullet$	1.98×10^{8} M^{-1} s^{-1}
$CH_3CH_2O\bullet + H\bullet \rightarrow CH_4 + CH_2O$	3.48×10^{3} M^{-1} s^{-1}
$CH_3CH_2O\bullet + H\bullet \rightarrow CH_3CH_2OH$	6.75×10^{3} M^{-1} s^{-1}
$(CH_3)_2CO + \bullet CH_3 \rightarrow (CH_3)_3CO\bullet$	6.62×10^{6} M^{-1} s^{-1}
$(CH_3)_3CO\bullet + CH_3CHO \rightarrow (CH_3)_3COH + CH_3CO\bullet$	4.85×10^{3} s^{-1}
$CH_3CO\bullet + H_2 \rightarrow CH_3CHO + H\bullet$	1.60×10^{-5} M^{-1} s^{-1}
$(CH_3)_3CO\bullet + CO \rightarrow CO_2 + (CH_3)_3C\bullet$	0.24 M^{-1} s^{-1}
$(CH_3)_2CO + (CH_3)_3CO\bullet \rightarrow (CH_3)_3COH + CH3CH_2CO$	3.95×10^{5} M^{-1} s^{-1}
$H\bullet + CH_3CHO \rightarrow CH_3CH_2O\bullet$	1.62×10^{5} M^{-1} s^{-1}
$H\bullet + CH_3CHO \rightarrow H_2 + CH_3CO\bullet$	4.05×10^{7} M^{-1} s^{-1}
$H\bullet + CH_3CHO \rightarrow H_2 + CO + \bullet CH_3$	5.75×10^{7} M^{-1} s^{-1}
$\bullet CH_3 + CH_3CHO \rightarrow CH_4 + CH_3CO\bullet$	3.28×10^{3} M^{-1} s^{-1}

$\bullet CH_3 + CH_3CHO \rightarrow H\bullet + CH_3CO\bullet$	$1.34 \times 10^{-2}\ M^{-1}\ s^{-1}$
$H\bullet + (CH_3)_3COH \rightarrow (CH_3)_3C\bullet + H_2O$	$3.83 \times 10^{4}\ M^{-1}\ s^{-1}$
$\bullet OH + (CH_3)_3COH \rightarrow (CH_3)_3CO\bullet + H_2O$	$1.04 \times 10^{8}\ M^{-1}\ s^{-1}$
$CH_3CH_2OH + H\bullet \rightarrow CH_3CH_2\bullet + H_2O$	$1.79 \times 10^{6}\ M^{-1}\ s^{-1}$
$CH_3CH_2OH + H\bullet \rightarrow CH_3CHOH\bullet + H_2$	$1.81 \times 10^{6}\ M^{-1}\ s^{-1}$
$CH_3CH_2OH + H\bullet \rightarrow \bullet CH_2CH_2OH + H_2$	$1.68 \times 10^{3}\ M^{-1}\ s^{-1}$
$CH_3CH_2OH + H\bullet \rightarrow CH_3CH_2O\bullet + H_2$	$26.0\ M^{-1}\ s^{-1}$
$H\bullet + CH_2O \rightarrow \bullet CHO + H_2$	$3.44 \times 10^{7}\ M^{-1}\ s^{-1}$
$H\bullet + \bullet CHO \rightarrow CO + H_2$	$6.80 \times 10^{10}\ M^{-1}\ s^{-1}$
$\bullet CHO + \bullet CHO \rightarrow CH_2O + CO$	$2.70 \times 10^{10}\ M^{-1}\ s^{-1}$
$\bullet CHO + \bullet CH_3 \rightarrow CH_3CHO$	$2.66 \times 10^{10}\ M^{-1}\ s^{-1}$
$\bullet CHO + \bullet CH_3 \rightarrow CH_4 + CO$	$2.65 \times 10^{10}\ M^{-1}\ s^{-1}$
$\bullet CH_2OH + H\bullet \rightarrow CH_2O + H_2$	$3.00 \times 10^{10}\ M^{-1}\ s^{-1}$
$\bullet CH_2OH + CHO\bullet \rightarrow CH_2O + CH_2O$	$1.80 \times 10^{11}\ M^{-1}\ s^{-1}$
$\bullet CH_2OH + \bullet CH_3 \rightarrow CH_2O + CH_4$	$8.49 \times 10^{10}\ M^{-1}\ s^{-1}$
$CH_3OH + \bullet CH_3 \rightarrow \bullet CH_2OH + H_2$	$1.74 \times 10^{11}\ M^{-1}\ s^{-1}$
$\bullet CH_2OH + \bullet H \rightarrow CH_2O + H_2$	$7.64 \times 10^{5}\ M^{-1}\ s^{-1}$
$CH_3OH + \bullet H \rightarrow CH_3O\bullet + H_2$	$11.0\ M^{-1}\ s^{-1}$
$CH_3OH + \bullet CH_3 \rightarrow CH_4 + \bullet CH_2OH$	$16.0\ M^{-1}\ s^{-1}$
$CH_3OH + \bullet CH_3 \rightarrow CH_4 + CH_3O\bullet$	$6.08\ M^{-1}\ s^{-1}$
$CH_3O\bullet + H\bullet \rightarrow CH_2O + H_2$	$1.99 \times 10^{10}\ M^{-1}\ s^{-1}$
$CH_3O\bullet + H\bullet \rightarrow CH_3OH$	$2.04 \times 10^{11}\ M^{-1}\ s^{-1}$
$CH_3O\bullet + CH_3CO\bullet \rightarrow CH_2O + CH_3CHO$	$3.42 \times 10^{10}\ M^{-1}\ s^{-1}$
$CH_3O\bullet + \bullet CH_3 \rightarrow CH_2O + CH_4$	$2.71 \times 10^{10}\ M^{-1}\ s^{-1}$
$CH_3O\bullet + CO \rightarrow \bullet CH_3 + CO$	$39.5\ M^{-1}\ s^{-1}$
$CH_3CHO + CH_3O\bullet \rightarrow CH_3OH + CH_3CO\bullet$	$5.00 \times 10^{6}\ M^{-1}\ s^{-1}$
$CH_3O\bullet + CH_4 \rightarrow CH_3OH + \bullet CH_3$	$56.7\ M^{-1}\ s^{-1}$
$CH_3O\bullet + CH_2O \rightarrow CH_3OH + \bullet CHO$	$686.51\ M^{-1}\ s^{-1}$
$\bullet CH_2OH + \bullet CH_2OH \rightarrow (CH_2OH)_2$	$1.8\times 10^{9}\ M^{-1}\ s^{-1}$
$CH_3OH + e^{-}_{s} \rightarrow CH_3O^{-} + H\bullet$	$1.00 \times 10^{4}\ M^{-1}\ s^{-1}$
$CH_3CH_2OH + e^{-}_{s} \rightarrow CH_3CH_2O^{-} + H\bullet$	$1.00 \times 10^{4}\ M^{-1}\ s^{-1}$

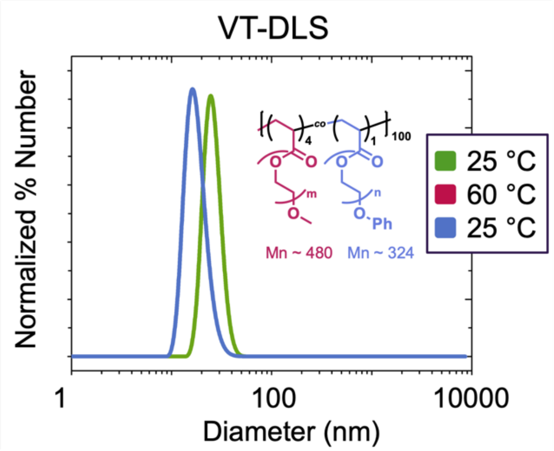

Supplementary Figure 1. VT-DLS of PEGMeA-*co*-PEGPhA in IPA. Note, no signal is observed at 60 °C given the complete solubility of the polymer at this temperature.

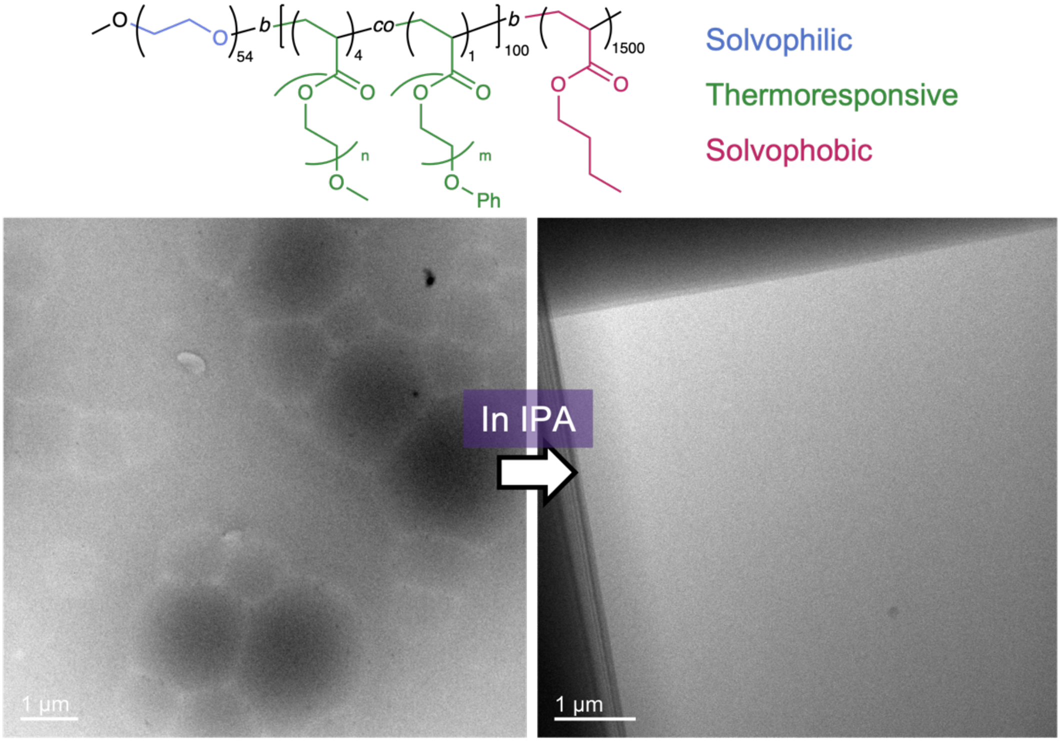

Supplementary Figure 2. Characterization of PEG-*b*-PEGMeA-*co*-PEGPhA-*b*-PnBA polymer. On the left, in dry state, the polymer is only visible through coffee-ring drying artifacts. On the right, the polymer is shown in liquid IPA at a flux of 0.33 e^{-} $Å^{-2}$ s^{-1}.

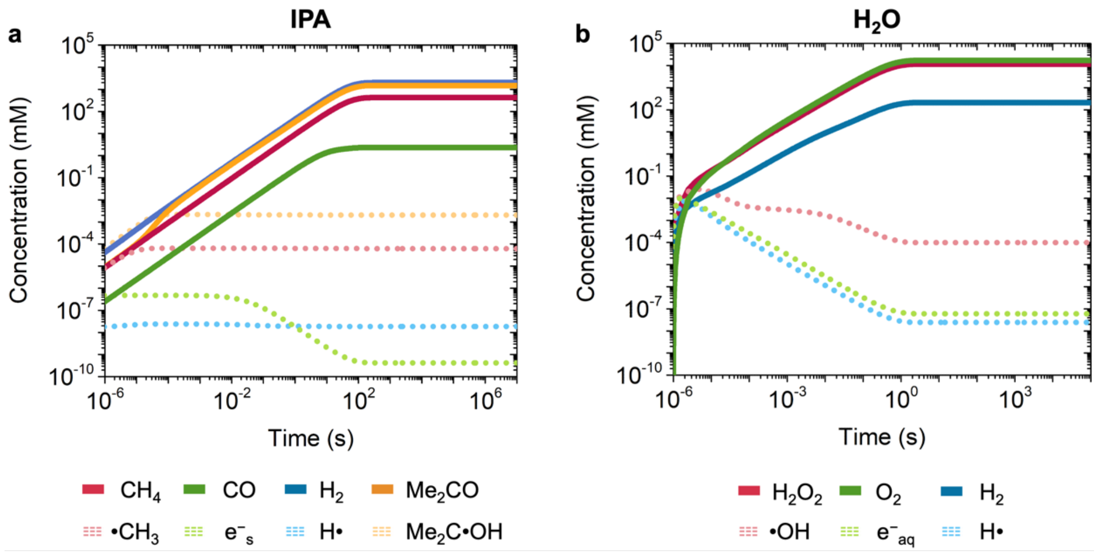

Supplementary Figure 3. Modeled results for solvent radiolysis using the reaction engineering module in COMSOL. Results for **a.** isopropanol and **b.** water.[1] For all modeling, a 300 keV electron beam with a beam current of 1 nA and radius of 1 μm were used.

To guide LCTEM studies on the isopropanol-based UCST nano-assemblies, we first endeavored to gain insight into the radiolysis of isopropanol (IPA). To do so, we developed a kinetic model for the radiolysis of isopropanol under LCTEM conditions. Mirroring previous studies,[1, 59] we assumed that all radiolytic species are homogeneously distributed in the irradiated solvent and that the concentration of the solvent remains constant. The concentration of each radiolytic species was calculated by summing the rates of individual reactions between different radiolytic species and the yield due to direct irradiation. We note that over 60 species are formed from the radiolysis of IPA so we have chosen to focus on the most reactive and dominant species to easily compare the radiolysis of IPA to that of water (**Supplementary Figure 3**, **Supplementary Tables 1-2**).[1]

There are several distinctions between the radiolysis of IPA and water. For radicals formed in both solvents, notably hydrogen atoms (H•) and solvated electrons (e_s^- or e_{aq}^-), a lower steady state concentration is observed in IPA compared to water (**Supplementary Figure 3a-b**). This can be rationalized by the formation of acetone (Me_2CO), a solvated electron scavenger, and methanol (MeOH), ethanol (EtOH), and

t-butanol (t-BuOH), all hydrogen atom scavengers (**Supplementary Table 1**).[60] Furthermore, hydroxyl radicals (•OH) formed in water and methyl radicals (•CH_3) formed in IPA both approach a steady state concentration of roughly 10^{-4} mM, but given the instability of radicals centered on more electronegative atoms, •OH radicals formed in water are more reactive than •CH_3 radicals formed in IPA.[7, 61] Thus, compared to •CH_3, •OH radicals formed in water are likely to cause more secondary damage to polymeric samples under investigation, as observed in previous studies.[62, 63, 64, 65, 66] The isopropyl radical (Me_2C•OH) formed from IPA radiolysis, though attaining the highest state concentration (~10^{-2} mM) of the examined radical species, is the most stable and thus least reactive radical formed in either solvent. Overall, the comparatively low reactivity and low steady state concentrations of radicals formed in IPA suggests that IPA is more amenable to LCTEM studies than water. Moreover, the lower density of IPA compared to water should allow for improved visibility of low contrast, solution-phase organic materials, making LCTEM uniquely capable of studying such samples.

II. Additional Data

For LCTEM damage experiments performed on proxy polymers, we used MALDI-IMS to create mass-filtered maps of the liquid-cell chip surfaces (**Supplementary Figure 4**). From this data, we obtained average mass spectra of the liquid-cell chip surfaces, which we then used to assess the MALDI mass signal intensity of the sample under different imaging conditions (**Supplementary Figure 5**). As revealed by MALDI-IMS, PEG-*b*-PEGMeA-*co*-PEGPhA and PEGMeA remained relatively intact under low flux conditions and showed reduced I_s/I_c values under high flux conditions, indicating enhanced sample degradation under higher flux conditions (**Figure 2b, Supplementary Figures 4-6**).[62] Interestingly, the PDEGEA polymer showed similar signal intensity under low and high flux conditions, suggesting that damage to the OEG sidechains is the dominant form of electron beam damage for these acrylic polymers, rather than damage to the polymer backbone. Since degradation of the OEG sidechains should reduce the solvophilicity of the polymer, damage to these sidechains could be a potential cause of the observed film

formation under continuous LCTEM imaging (**Figure 2a**). Likewise, as PS showed enhanced degradation under continuous imaging, crosslinking of PS polymer chains could also increase solvophobicity and thus contribute to the observed film formation. Ultimately, our MALDI-IMS studies suggest that the triblock copolymer likewise survives under low flux conditions, whereas under high flux conditions, the particle expansion is a manifestation of sample damage (**Figures 2a, 3a**).

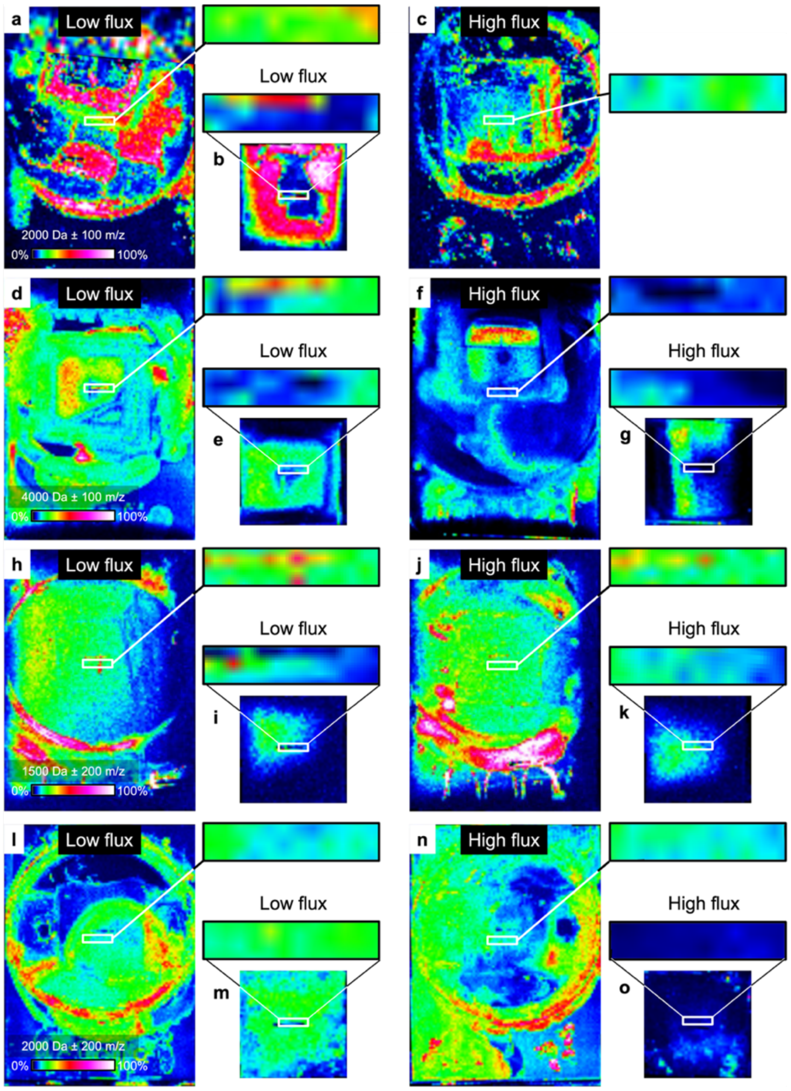

Supplementary Figure 4. MALDI-IMS colormaps analysis of liquid-cell chip surfaces. Low flux LCTEM experiments were performed via stroboscopic imaging at a flux of 0.33 e^{-} $Å^{-2}$ s^{-1} and fluence of < 2 e^{-} $Å^{-2}$. High flux LCTEM experiments were performed via continuous imaging at a flux of 1.42 e^{-} $Å^{-2}$

s^{-1} and fluence of 15000 e^{-} Å^{-2}. MALDI-IMS post-mortem colormap of top and bottom chip for **a-b.** low flux and **c.** top chip for high flux LCTEM experiments on PEG-*b*-PEGMeA-*co*-PEGPhA with a mass filter of 2000 ± 100 m/z. Note, small chip was broken and thus not analyzed. MALDI-IMS post-mortem colormap of top and bottom chip for **d-e.** low flux and **f-g.** high flux LCTEM experiments on PEGMeA with a mass filter of 4000 ± 100 m/z. MALDI-IMS post-mortem colormap of top and bottom chip for **h-i.** low flux and **j-k.** high flux LCTEM experiments on PDEGEA with a mass filter of 1500 ± 200 m/z. MALDI-IMS post-mortem colormap of top and bottom chip for **l-m.** low flux and **n-o.** high flux LCTEM experiments on PS with a mass filter of 2000 ± 100 m/z. All data is shown with a mass filter displayed as 0–100% of total intensity on a logarithmic scale.

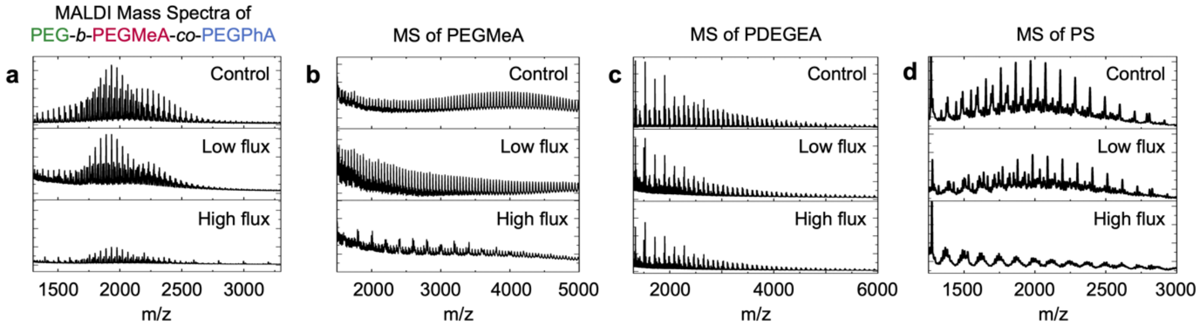

Supplementary Figure 5. MALDI-IMS mass spectra for corresponding colormaps shown in Supplementary Figure 4. Control samples were deposited on liquid-cell chip and not imaged. MALDI-IMS control low flux, and high flux mass spectra for **a.** PEG-*b*-PEGMeA-*co*-PEGPhA, **b.** PEGMeA, **c.** PDEGEA, **d.** PS.

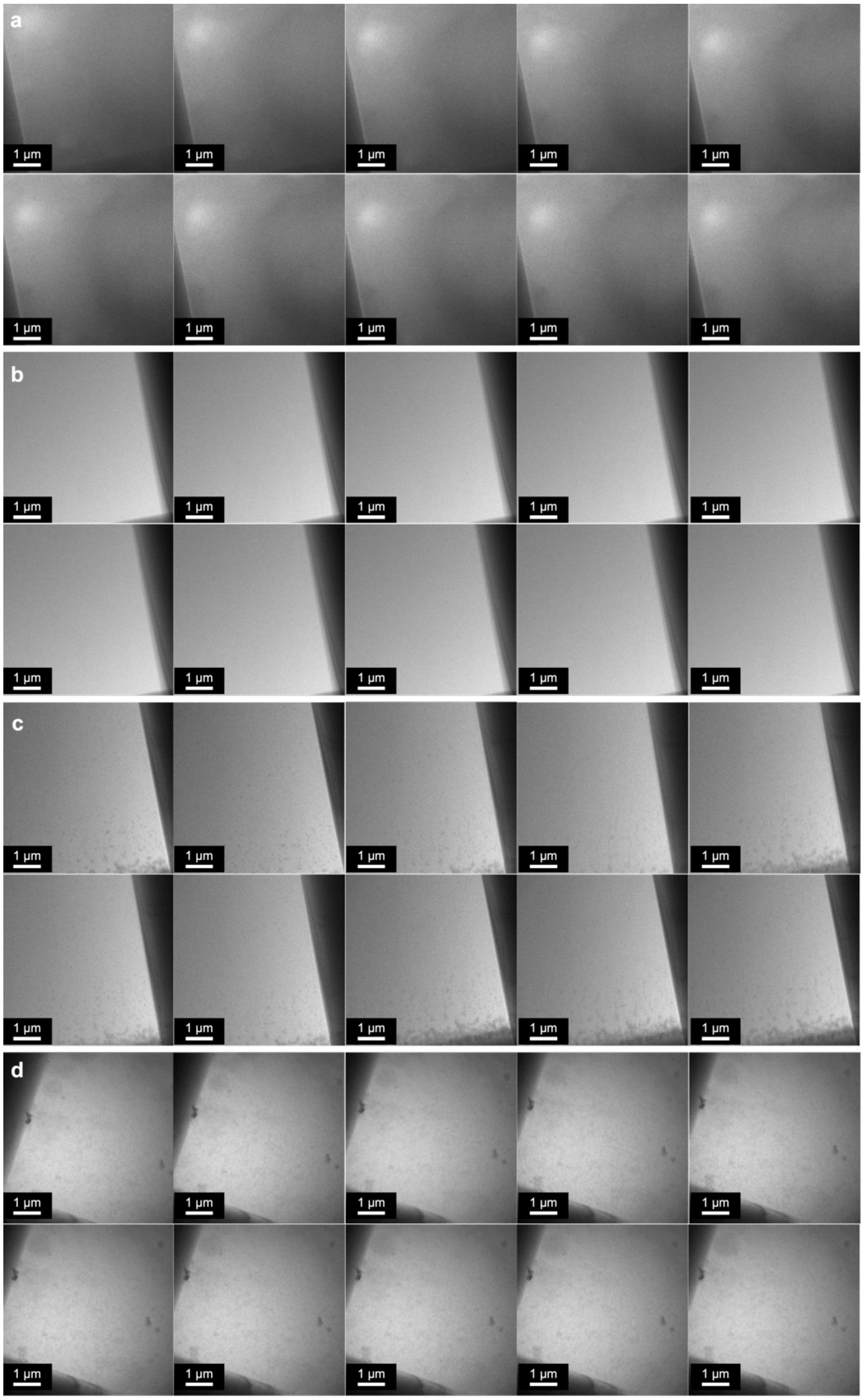

Supplementary Figure 6. LCTEM damage experiments. The experiments were performed via stroboscopic imaging at a flux of 0.33 e^{-} $Å^{-2}$ s^{-1} and fluence of < 2 e^{-} $Å^{-2}$ on **a.** PEG-*b*-PEGMeA-*co*-PEGPhA, **b.** PEGMeA, **c.** PDEGEA, **d.** PS.

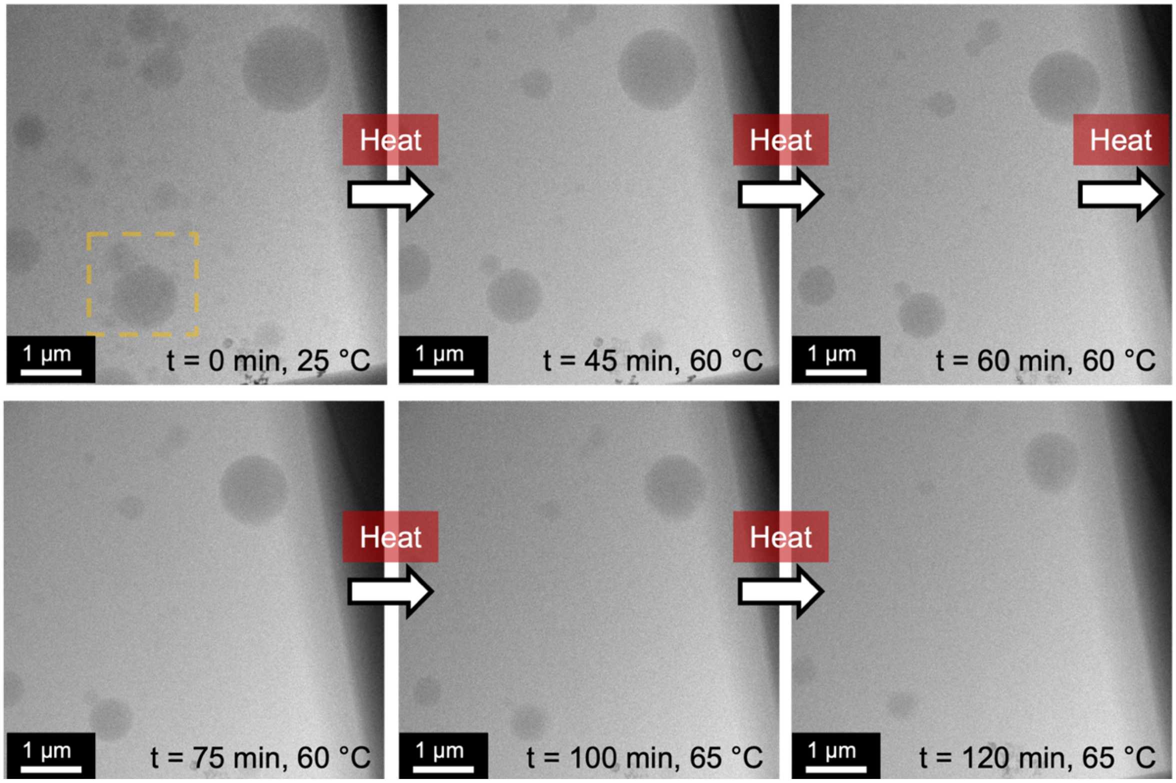

Supplementary Figure 7. VT-LCTEM experiment on PEG-*b*-PEGMeA-*co*-PEGPhA-*b*-PS in IPA imaged at a flux of 0.33 e^{-} $Å^{-2}$ s^{-1} and heated to 65 °C. Uncropped dataset of regions of interest shown in Figure 2 of main text

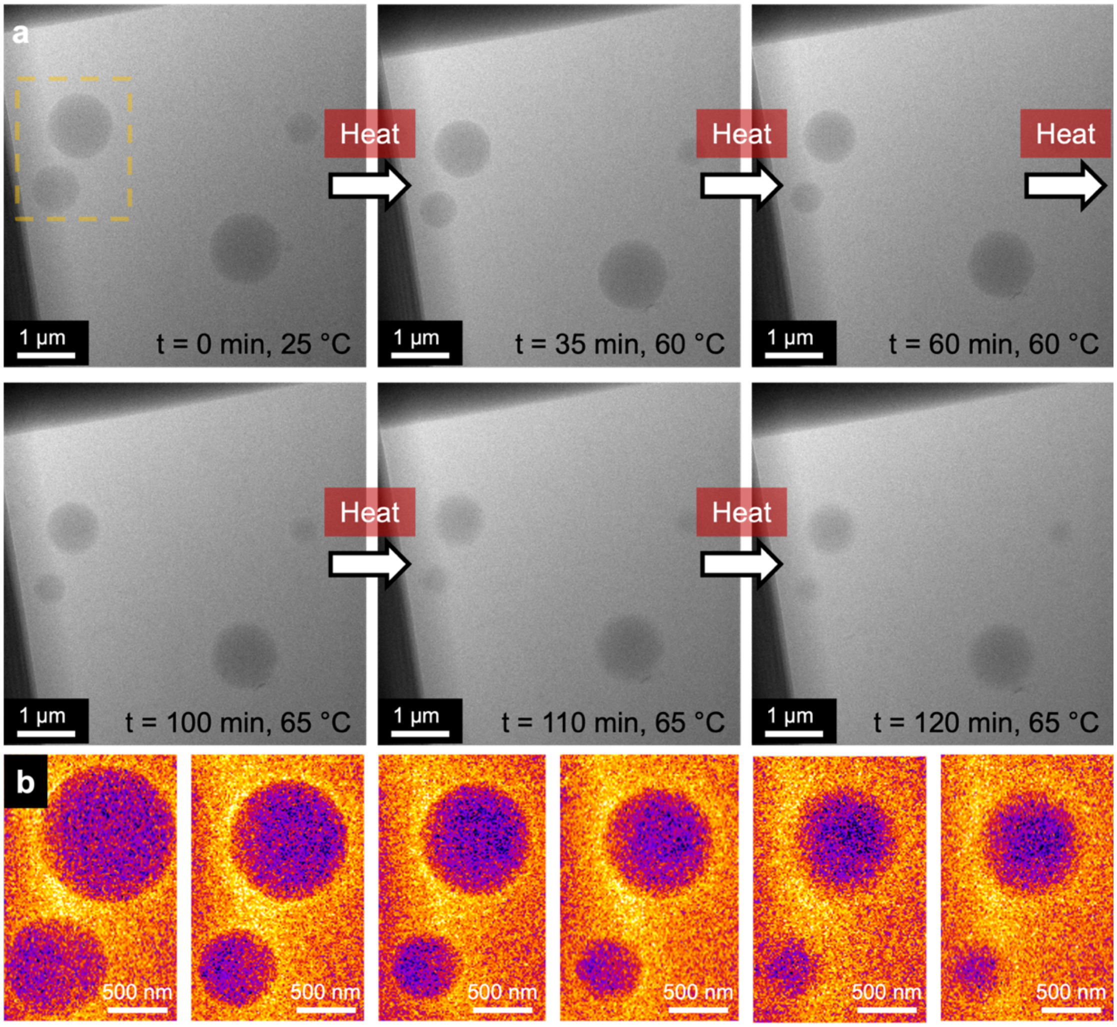

Supplementary Figure 8. VT-LCTEM experiment on PEG-*b*-PEGMeA-*co*-PEGPhA-*b*-PS in IPA imaged at a flux of 0.33 e^{-} $Å^{-2}$ s^{-1} and heated to 65 °C. a. Raw data and **b.** false-colored, processed data. Repeat of experiment shown in Figure 2 of main text.

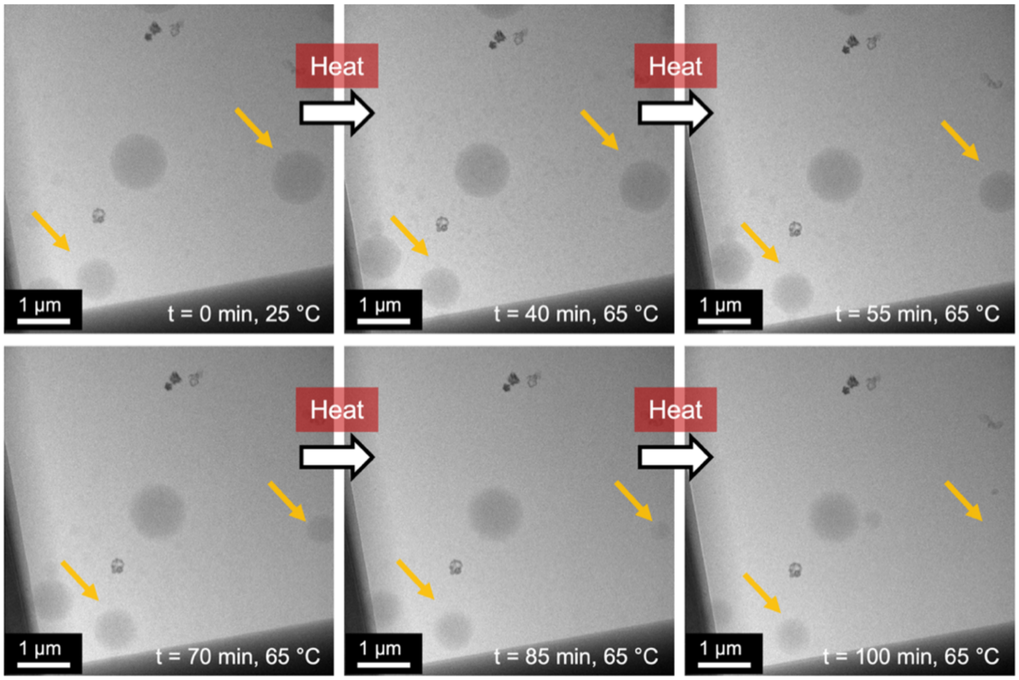

Supplementary Figure 9. VT-LCTEM experiment on PEG-*b*-PEGMeA-*co*-PEGPhA-*b*-PS in IPA imaged at a flux of 0.33 e^{-} Å^{-2} s^{-1} and heated to 65 °C. Repeat of experiment shown in Figure 2 of main text.

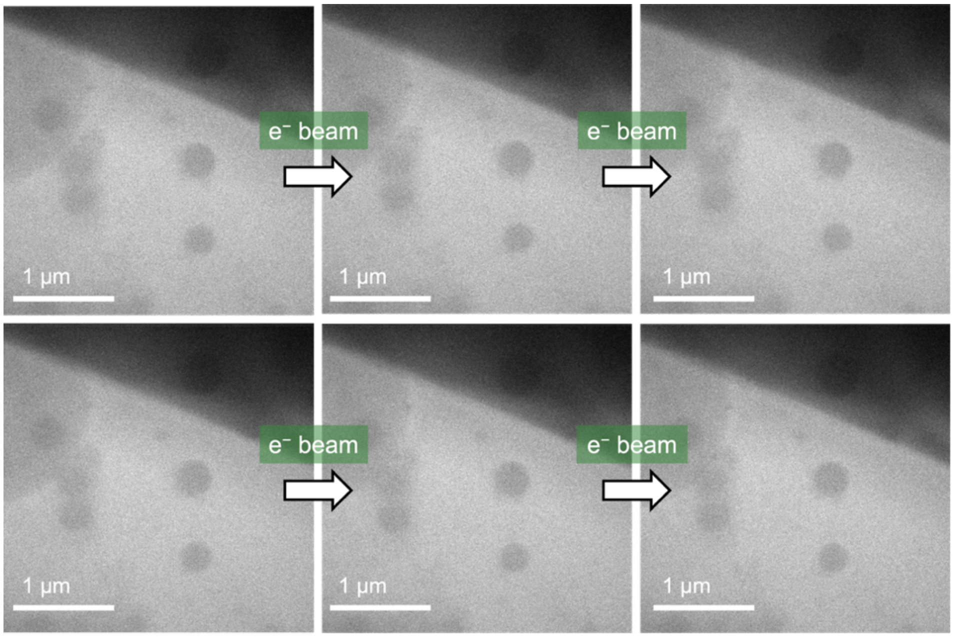

Supplementary Figure 10. Control LCTEM experiment imaged at 0.33 e^{-} Å^{-2} s^{-1}. Control experiment of unheated UCST polymer showing electron beam dose not trigger particle size reduction.

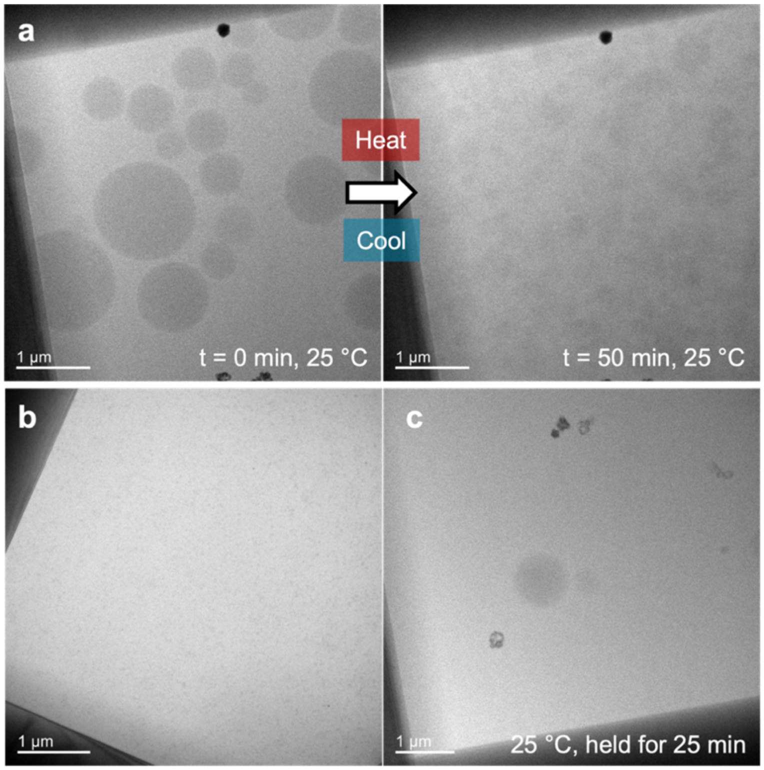

Supplementary Figure 11. Control LCTEM experiments all imaged at 0.33 e^{-} Å^{-2} s^{-1}. a. Heating and cooling cycle VT-LCTEM experiment showing the irreversibility of the UCST transformation. **b.** Post-

mortem dry state of LC chip showing uniform small assemblies **c.** Final, cooled timepoint of data shown in Supplementary Figure 3 showing irreversibility of the UCST transformation.

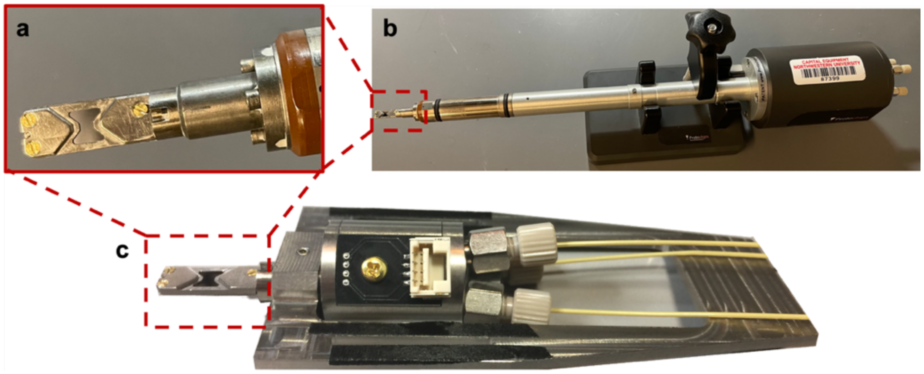

Supplementary Figure 12. LC holder design. a. Liquid-cell holder tip common to both LCTEM and RSoXS. **b.** Full view of LCTEM holder. **c.** Full view of RSoXS holder.

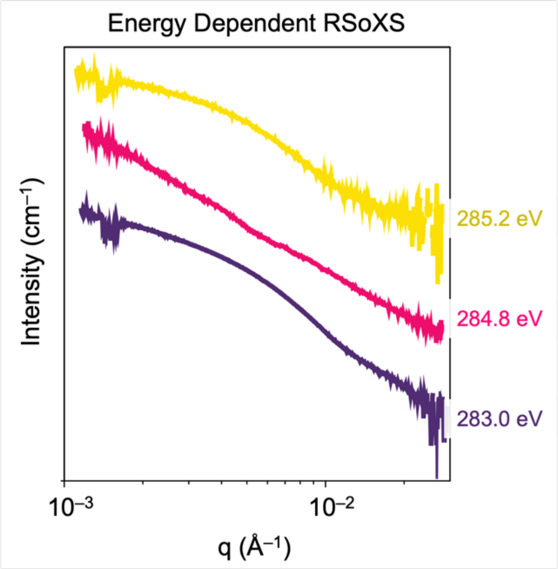

Supplementary Figure 13. RSoXS data measured for a solution of PEG-*b*-PEGMeA-*co*-PEGPhA and PEGMeA in IPA. Data was measured before heating using energies of 285.2 eV, 284.8 eV, and 283.0 eV to determine best energy for subsequent measurements.

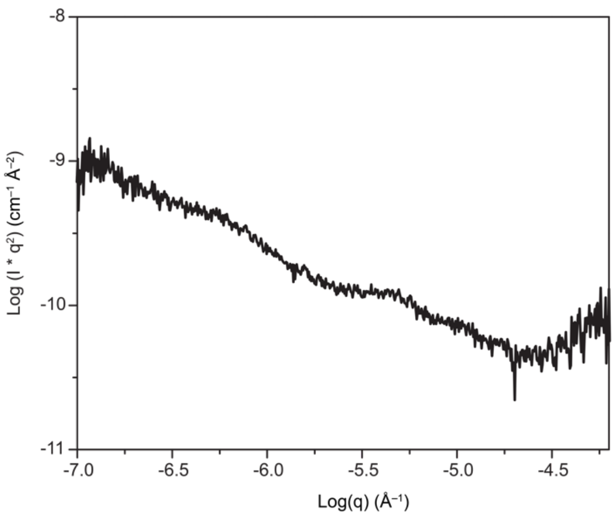

Supplementary Figure 14. RSoXS data measured for a solution of PEG-*b*-PEGMeA-*co*-PEGPhA and PEGMeA in IPA. Data is shown in log($I \times q^2$) vs ln q plot for 285.2 eV.

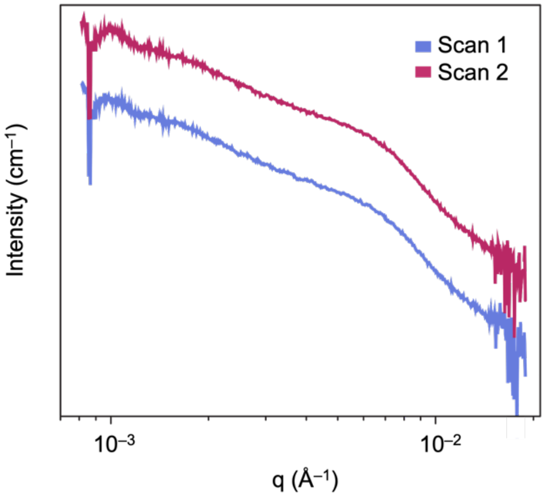

Supplementary Figure 15. Two RSoXS traces measured for a solution of PEG-*b*-PEGMeA-*co*-PEGPhA and PEGMeA in IPA at 283 eV after the heating and cooling cycle. This data highlights that upon subsequent exposures, the sample does not undergo visible beam induced degradation.

Description of X-Ray Scattering Models

The 1D scattering intensity for the polymer micelle model is calculated according to the equations given by Pedersen.[67, 68] In the following discussion, we reproduce the description for the polymer micelle model

compiled by SASView. The micelle core of radius r is represented by N polymer heads, each of volume V_{core}. Gaussian random coil tails, with a radius of gyration R_g, are distributed around the spherical core. These coils are centered at a distance of $r + d{\cdot}R_g$ from the micelle center. Here, d is a penetration term on the order unity. For each coil, a volume of V_{corona} can be defined.

$$P(q) = N^2\beta_s^2\Phi(qr)^2 + N\beta_c^2P_c(q) + 2N^2\beta_s\beta_cS_{sc}(q) + N(N-1)\beta_c^2S_{cc}(q) \quad (4)$$

$$\beta_s = V_{core}(\rho_{core} - \rho_{solvent}) \quad (5)$$

$$\beta_c = V_{corona}(\rho_{corona} - \rho_{solvent}) \quad (6)$$

where ρ_{corona}, $\rho_{solvent}$, ρ_{core} are the scattering length densities. For the spherical core of radius r

$$\Phi(qr) = \frac{sin(qr) - qrcos(qr)}{(qr)^3} \quad (7)$$

And for the Gaussian coils

$$P_c(q) = 2[exp(-Z) + Z-1] / Z^2 \quad (8)$$

$$Z = (qR_g)^2 \quad (9)$$

The core-to-corona and corona-to-corona cross terms are approximated by:

$$S_{sc}(q) = \frac{\Phi(qr)\psi(Z)sin(q(r+d{\cdot}R_g))}{q(r+d{\cdot}R_g)} \quad (10)$$

$$S_{cc}(q) = \left[\frac{sin(q(r+d{\cdot}R_g))}{q(r+d{\cdot}R_g]}\right]^2 \quad (11)$$

$$\psi(Z) = \frac{1-exp^{-Z}}{Z} \quad (12)$$

Supplementary Table 3. Fixed fitting parameters for all 283 eV traces using a spherical polymer micelle model.

**Parameter**	**Value**
Scale	0.02
Background	0.02
Number Density	2866E+15 cm^{-1}
Core Volume	7.7962E+05 $Å^3$
Corona Volume	1.1319E+07 $Å^3$
Solvent SLD	1.84E-6 $Å^{-2}$
Core SLD	-1.54E-6 $Å^{-2}$
Corona SLD	1.5E-6 $Å^{-2}$
Penetration Factor	0.9158
Aggregation Number	2.535

Supplementary Table 4. Core radii and radii of gyration (R_g) for coronal chains measured at 283 eV as a function of temperature and time.

**Measurement**	**Core radius**	**R_g of coronal chains**
25 °C, 0 min	47 nm	48 nm
60 °C, 10 min	42 nm	59 nm
60 °C, 15 min	39 nm	73 nm
60 °C, 30 min	38 nm	75 nm
< 35 °C, 30 min	38 nm	65 nm

For the sticky hard sphere model,[68, 69, 70, 71] the perturbation parameter, τ, should be fixed between 0.01 and 0.1. The stickiness, ϵ, is used to adjust the interaction strength. The stickiness is a function of both the perturbation parameter and the interaction strength. ϵ and τ are defined in terms of the hard sphere diameter ($\sigma = 2R$), the width of the square well, Δ, and the depth of the well, U_o.

$$\epsilon = (1/12\tau) \exp (U_o / kT) \quad (13)$$

$$\tau = \Delta / (\sigma + \Delta) \quad (14)$$

where the interaction potential is

$$U(r) = \begin{cases} \infty & r < \sigma \\ -U_o & \sigma \leq r \leq \sigma + \Delta \\ 0 & r > \sigma + \Delta \end{cases} \quad (15)$$

Supplementary Table 5. Fixed fitting parameters for all 285.2 eV trace using a sphere with a sticky hard sphere form factor.

Parameter	Value
Scale	0.02
Background	0.02
Solvent SLD	1.35E-6 $Å^{-2}$
SLD	0.4E-6 $Å^{-2}$
Radius	207 nm
Volume Fraction	0.18
Perturbation	0.099
Stickiness	0.12

Supplementary Table 6. Fixed fitting parameters for all 288.8 eV trace using a sphere with a sticky hard sphere form factor.

Parameter	Value
Scale	0.02
Background	0.25
Solvent SLD	3.76E-6 $Å^{-2}$
SLD	3.32E-6 $Å^{-2}$
Radius	650 nm
Volume Fraction	0.14
Perturbation	0.099
Stickiness	0.12
Distribution of radius (gaussian)	0.807

III. Synthesis

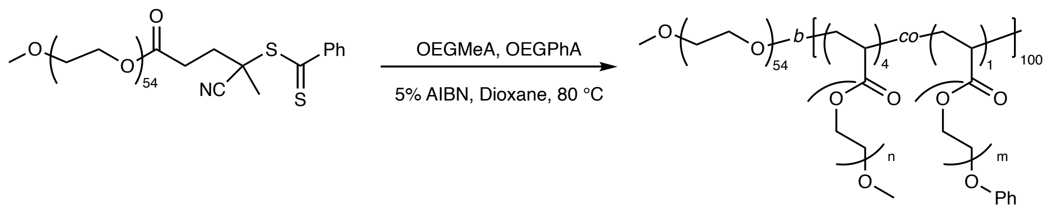

Supplementary Figure 16. Synthesis of PEG-*b*-PEGMeA-*co*-PEGPhA. The polymer was prepared via reversible addition fragmentation chain transfer.

To a 20 mL glass vial equipped with a septum, 2 kDa poly(ethylene glycol) 4-cyano-4-(phenylcarbonothioylthio)pentanoate (PEG, 46.6 mg, 0.019 mmol), azobisisobutyronitrile (AIBN, 0.16 mg, 0.97 µmol), poly(ethylene glycol) methyl ether acrylate (OEGMeA, 684 µL, 1.33 mmol), poly(ethylene glycol) phenyl ether acrylate (OEGPhA, 119 µL, 0.33 mmol), and 1,4-dioxane (2000 µL) were added. N_2 gas was bubbled through the solution for 2 hours before the vial was heated in an oil bath to 80 °C for 3 hours under stirring. The reaction was quenched by submerging the reaction mixture in ice water.

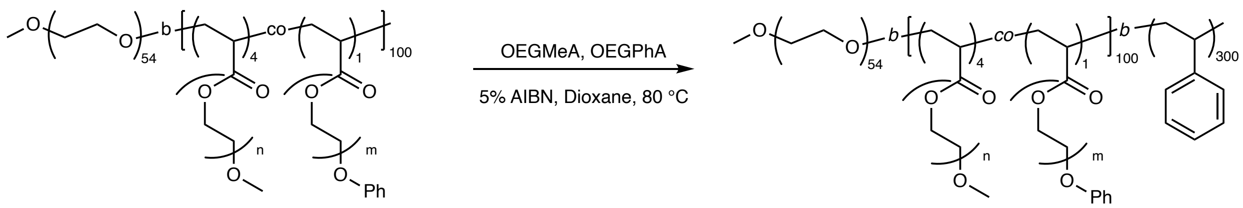

Supplementary Figure 17. Synthesis of PEG-*b*-PEGMeA-*co*-PEGPhA-*b*-PS. The polymer was prepared via reversible addition fragmentation chain transfer.

To a 20 mL glass vial equipped with a septum, PEG-*b*-PEGMeA-*co*-PEGPhA (36.8 mg), AIBN (0.09 mg, 0.54 µmol), styrene (St, 832 µL, 0.07 mmol), and isopropanol (IPA, 1700 µL) were added. N_2 gas was bubbled through the solution for 2 hours before the vial was heated in an oil bath to 80 °C for 19 hours under stirring. The reaction was quenched by submerging the reaction mixture in ice water.

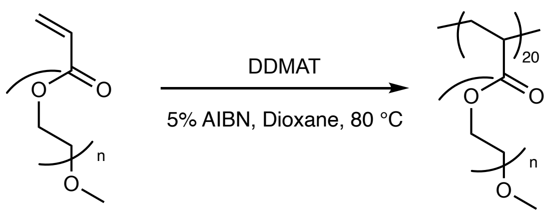

Supplementary Figure 18. Synthesis of PEGMeA. The polymer was prepared via reversible addition fragmentation chain transfer.

To a 20 mL glass vial equipped with a septum, OEGMeA (183 µL, 0.40 mmol), AIBN (0.13 mg, 0.80 µmol), DDMAT (5.86 mg, 0.02 mmol), and 1,4-dioxane (400 µL) were added. N_2 gas was bubbled through the solution for 2 hours before the vial was heated in an oil bath to 80 °C for 5 hours under stirring. The reaction was quenched by submerging the reaction mixture in ice water.

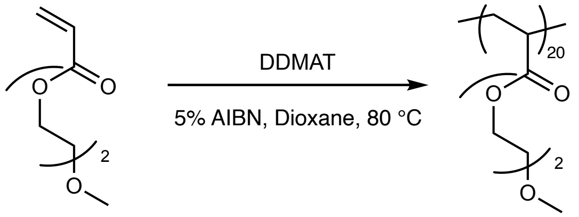

Supplementary Figure 19. Synthesis of PDEGEA. The polymer was prepared via reversible addition fragmentation chain transfer.

To a 20 mL glass vial equipped with a septum, di(ethylene glycol) ethyl ether acrylate (DEGEA) (477 µL, 2.66 mmol), AIBN (0.43 mg, 2.70 µmol), DDMAT (19.41 mg, 0.05 mmol), and 1,4-dioxane (900 µL) were added. N_2 gas was bubbled through the solution for 2 hours before the vial was heated in an oil bath to 80 °C for 5 hours under stirring. The reaction was quenched by submerging the reaction mixture in ice water.

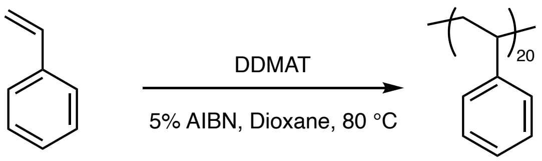

Supplementary Figure 20. Synthesis of PS. The polymer was prepared via reversible addition fragmentation chain transfer.

To a 20 mL glass vial equipped with a septum, styrene (173 µL, 1.51 mmol), AIBN (0.41 mg, 2.5 µmol), DDMAT (18.32 mg, 0.05 mmol), and 1,4-dioxane (420 µL) were added. N_2 gas was bubbled through the solution for 2 hours before the vial was heated in an oil bath to 80 °C for 19 hours under stirring. The reaction was quenched by submerging the reaction mixture in ice water.

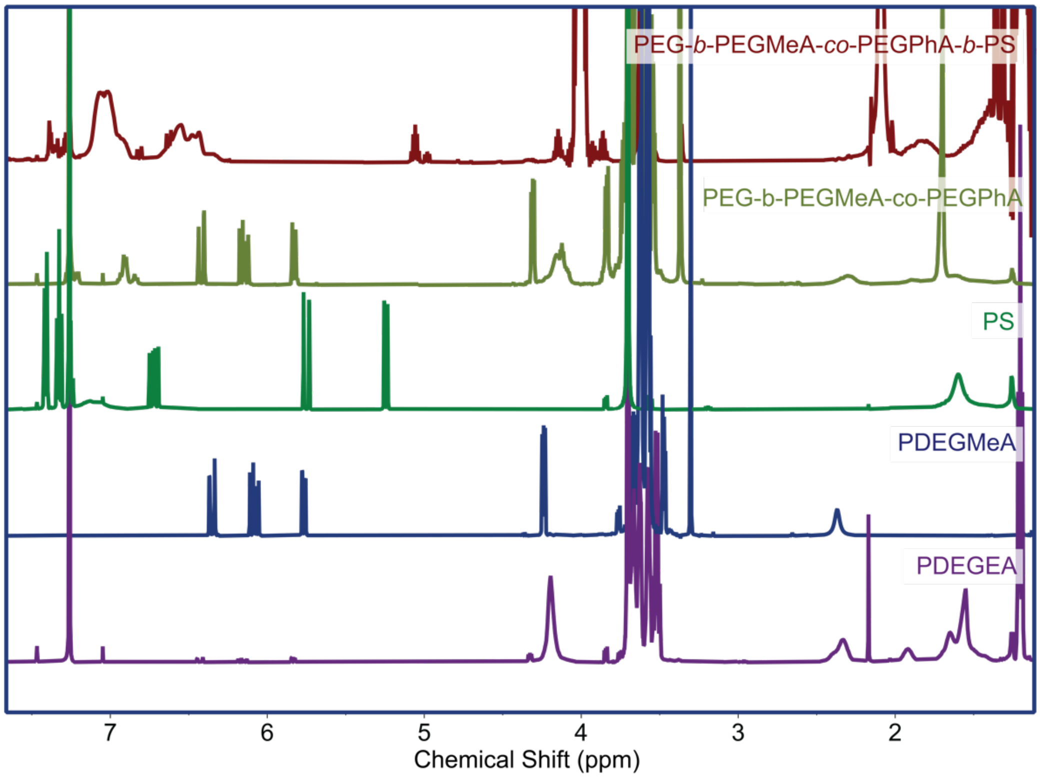

Supplementary Figure 21. Raw ^{1}H NMR (Proton Nuclear Magnetic Resonance) data. From top to bottom, the spectra for the PEG-*b*-PEGMeA-*co*-PEGPhA-*b*-PS, PEG-*b*-PEGMeA-*co*-PEGPhA, PS, PEGMeA, and PDEGEA polymers are shown.

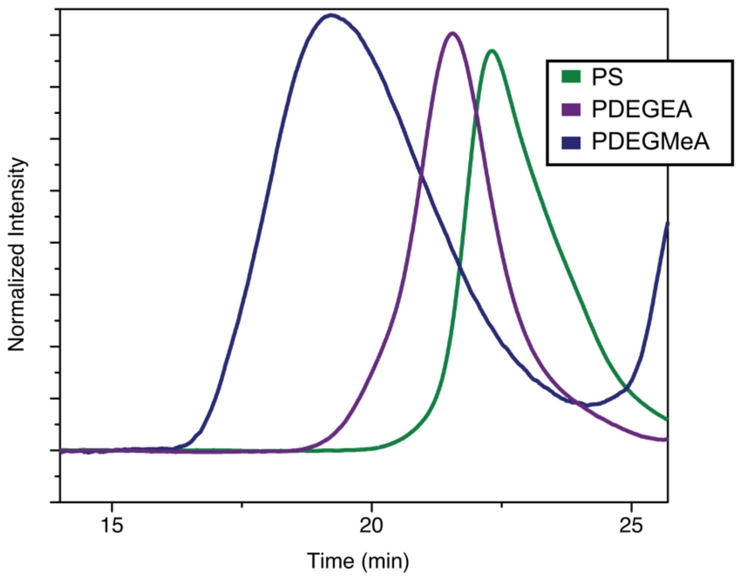

Supplementary Figure 22. Size exclusion chromatography multiangle light scattering (SEC-MALS) traces. Here, the data is shown for PS, PEGMeA, and PDEGEA. Data for PEG, PEG-*b*-PEGMeA-*co*-PEGPhA, and PEG-*b*-PEGMeA-*co*-PEGPhA-*b*-PS are shown in Figure 1.

Supplementary Table 7. SEC-MALS results for the examined polymers. The dn/dc was estimated to be 0.16 for PS and 0.08 for all synthesized polymers.[72]

**Polymer**	**M_n**	**M_w**	**Đ**
PEG-*b*-PEGMeA-*co*-PEGPhA-*b*-PS	1.477×10^5	1.755×10^5	1.188
PEG-*b*-PEGMeA-*co*-PEGPhA	2.553×10^4	3.069×10^4	1.192
PEGMeA	3.581×10^4	4.467×10^4	1.247
PDEGEA	8.833×10^3	9.296×10^3	1.052
PS	7.960×10^3	8.550×10^3	1.074

IV. References

1. Schneider NM, Norton MM, Mendel BJ, Grogan JM, Ross FM, Bau HH. Electron–Water Interactions and Implications for Liquid Cell Electron Microscopy. *The Journal of Physical Chemistry C* **118**, 22373-22382 (2014).

2. Berger MJ, Coursey JS, Zucker MA, Chang J. NIST Standard Reference Database.) (2017).

3. Gowd EB, Koga T, Endoh MK, Kumar K, Stamm M. Pathways of cylindrical orientations in PS-b-P4VP diblock copolymer thin films upon solvent vapor annealing. *Soft Matter* **10**, 7753-7761 (2014).

4. O'Driscoll S*, et al.* The morphology and structure of PS-b-P4VP block copolymer films by solvent annealing: effect of the solvent parameter. *Polymers for Advanced Technologies* **22**, 915-923 (2011).

5. Chatgilialoglu C, Crich D, Komatsu M, Ryu I. Chemistry of Acyl Radicals. *Chemical Reviews* **99**, 1991-2070 (1999).

6. Henry DJ, Parkinson CJ, Mayer PM, Radom L. Bond Dissociation Energies and Radical Stabilization Energies Associated with Substituted Methyl Radicals. *The Journal of Physical Chemistry A* **105**, 6750-6756 (2001).

7. Forrester AR, Hay JM, Thomson RH. Organic chemistry of stable free radicals. (1968).

8. Tamimi A, Rinker EB, Sandall OC. Diffusion coefficients for hydrogen sulfide, carbon dioxide, and nitrous oxide in water over the temperature range 293-368 K. *Journal of Chemical and Engineering data* **39**, 330-332 (1994).

9. Poudyal I, Adhikari NP. Temperature dependence of diffusion coefficient of carbon monoxide in water: A molecular dynamics study. *Journal of Molecular Liquids* **194**, 77-84 (2014).

10. Witherspoon P, Saraf D. Diffusion of Methane, Ethane, Propane, and n-Butane in Water from 25 to 43. *The Journal of Physical Chemistry* **69**, 3752-3755 (1965).

11. Moradi H, Azizpour H, Bahmanyar H, Mohammadi M, Akbari M. Prediction of methane diffusion coefficient in water using molecular dynamics simulation. *Heliyon* **6**, e05385 (2020).

12. Sørensen K, Finster K, Ramsing N. Thermodynamic and kinetic requirements in anaerobic methane oxidizing consortia exclude hydrogen, acetate, and methanol as possible electron shuttles. *Microbial Ecology* **42**, 1-10 (2001).

13. Chu D, Gilman S. The Influence of Methanol on O 2 Electroreduction at a Rotating Pt Disk Electrode in Acid Electrolyte. *Journal of the Electrochemical Society* **141**, 1770 (1994).

14. Bolton GL, Freeman GR. Solvated electron reaction rates in alcohols and water. Solvent effect. *Journal of the American Chemical Society* **98**, 6825-6830 (1976).

15. Barat F, Gilles L, Hickel B, Lesigne B. Effect of the dielectric constant on the reactivity of the solvated electron. *The Journal of Physical Chemistry* **77**, 1711-1715 (1973).

16. Hippler H, Striebel F, Viskolcz B. A detailed experimental and theoretical study on the decomposition of methoxy radicals. *Physical Chemistry Chemical Physics* **3**, 2450-2458 (2001).

17. Batt L, Burrows J, Robinson G. On the isomerisation of the methoxy radical relevance to atmospheric chemistry and combustion. *Chemical Physics Letters* **78**, 467-470 (1981).

18. Batt L, Robinson G. Reaction of methoxy radicals with oxygen. I. Using dimethyl peroxide as a thermal source of methoxy radicals. *International Journal of Chemical Kinetics* **11**, 1045-1053 (1979).

19. Lissi E, Massiff G, Villa A. Oxidation of carbon monoxide by methoxy-radicals. *Journal of the Chemical Society, Faraday Transactions 1: Physical Chemistry in Condensed Phases* **69**, 346-351 (1973).

20. Dainton FS, Salmon G, Wardman P. The radiation chemistry of liquid and glassy methanol. *Proceedings of the Royal Society of London A Mathematical and Physical Sciences* **313**, 1-30 (1969).

21. De Barros A, Domaracka A, Andrade D, Boduch P, Rothard H, Da Silveira E. Radiolysis of frozen methanol by heavy cosmic ray and energetic solar particle analogues. *Monthly Notices of the Royal Astronomical Society* **418**, 1363-1374 (2011).

22. Brimage D, Cassell J, Sharp J, Symons M. Unstable intermediates. Part LXV. Electron spin resonance studies of radicals derived from protonated alcohols by γ-irradiation: the radicals R 2 ĊOH 2+. *Journal of the Chemical Society A: Inorganic, Physical, Theoretical*, 2619-2621 (1969).

23. Hart EJ, Thomas J, Gordon S. A review of the radiation chemistry of single-carbon compounds and some reactions of the hydrated electron in aqueous solution. *Radiation Research Supplement* **4**, 74-88 (1964).

24. Marfak A, Trouillas P, Allais D-P, Champavier Y, Calliste C-A, Duroux J-L. Radiolysis of Quercetin in Methanol Solution: Observation of Depside Formation. *Journal of Agricultural and Food Chemistry* **50**, 4827-4833 (2002).

25. Öberg KI, Garrod RT, Van Dishoeck EF, Linnartz H. Formation rates of complex organics in UV irradiated CH3OH-rich ices-I. Experiments. *Astronomy & Astrophysics* **504**, 891-913 (2009).

26. Wargon JA, Williams E. Electron spin resonance studies of radical trapping in the radiolysis of organic liquids. I. Evidence for the primary formation of the methoxy radical in methanol. *Journal of the American Chemical Society* **94**, 7917-7918 (1972).

27. Ellison D, Salmon G, Wilkinson F. Nanosecond pulse radiolysis of methanolic and aqueous solutions of readily oxidizable solutes. *Proceedings of the Royal Society of London A Mathematical and Physical Sciences* **328**, 23-36 (1972).

28. Hayon E, Moreau M. Electron Capture by Solutes in the Radiolysis of Methanol and Ethanol. *The Journal of Physical Chemistry* **69**, 4053-4057 (1965).

29. Zhang G, Thomas JK. Pyrene Radical Formation in Pulse Radiolysis of Liquid Methanol. *The Journal of Physical Chemistry* **98**, 11714-11718 (1994).

30. Lind J, Jowko A, Eriksen TE. Methoxy radical production in methanol radiolysis. *Radiation Physics and Chemistry (1977)* **13**, 159-163 (1979).

31. Jha K, Freeman G. I. Kinetics of reactions of electrons during radiolysis of liquid methanol. II. Reaction of electrons with liquid alcohols and with water. *The Journal of Chemical Physics* **48**, 5480-5490 (1968).

32. Baxendale J, Sedgwick R. Radiolysis of methanol vapour. *Transactions of the Faraday Society* **57**, 2157-2166 (1961).

33. Cooper W, Tobien T. The application of the electron beam process in water and wastewater treatment: fundamental and applied studies.) (2001).

34. Monod A, Chebbi A, Durand-Jolibois R, Carlier P. Oxidation of methanol by hydroxyl radicals in aqueous solution under simulated cloud droplet conditions. *Atmospheric Environment* **34**, 5283-5294 (2000).

35. Huie RE, Clifton CL. Kinetics of the self-reaction of hydroxymethylperoxyl radicals. *Chemical physics letters* **205**, 163-167 (1993).

36. Baxendale JH, Wardman P. The radiolysis of methanol: product yields, rate constants, and spectroscopic parameters of intermediates.). NATIONAL STANDARD REFERENCE DATA SYSTEM (1975).

37. Urazbahtina LR, Remnev GE, Goncharov DV, Pushkarev AI. Radiolysis of methyl alcohol water solution by pulsed electron beam. In: *Proceedings. The 8th Russian-Korean International Symposium on Science and Technology, 2004. KORUS 2004.*) (2004).

38. Meaburn G, Mellows F, Reiffsteck A. Production of hydrogen in the radiolysis of methanol vapour. *Nature* **204**, 1301-1302 (1964).

39. Choi SU, Lichtin NN. The Radiolysis of Methanol and Methanolic Solutions. III. The Effect of Oxygen on the Radiolysis of Liquid Methanol by 60Co γ-Rays and by 10B (N, α) 7Li Recoils. *Journal of the American Chemical Society* **86**, 3948-3953 (1964).

40. Baxendale JH, Mellows FW. The γ-Radiolysis of Methanol and Methanol Solutions. *Journal of the American Chemical Society* **83**, 4720-4726 (1961).

41. Johnson DW, Salmon GA. Pulse radiolysis of methanol and ethanol. Acid-base behaviour of hydroxymethyl and hydroxyethyl radicals. *Journal of the Chemical Society, Faraday Transactions 1: Physical Chemistry in Condensed Phases* **71**, 583-591 (1975).

42. Meshitsuka G, Burton M. Radiolysis of Liquid Methanol by Co 60 Gamma-Radiation. *Radiation research* **8**, 285-297 (1958).

43. Dainton FS, Janovský I, Salmon G. The radiation chemistry of liquid methanol. I. The oxidizing radical. *Proceedings of the Royal Society of London A Mathematical and Physical Sciences* **327**, 305-316 (1972).

44. Getoff N, Ritter A, Schwörer F, Bayer P. Primary yields of CH3• O and• CH2OH radicals resulting in the radiolysis of high purity methanol. *Radiation Physics and Chemistry* **41**, 797-801 (1993).

45. Sullivan KK*, et al.* Low-energy (< 20 eV) and high-energy (1000 eV) electron-induced methanol radiolysis of astrochemical interest. *Monthly Notices of the Royal Astronomical Society* **460**, 664-672 (2016).

46. Getoff N, Ritter A, Schwörer F, Bayer P. Pulse radiolysis and product analysis of triethylsilane in methanol. *International Journal of Radiation Applications and Instrumentation Part C Radiation Physics and Chemistry* **39**, 177-182 (1992).

47. Hudson R, Moore M. Laboratory studies of the formation of methanol and other organic molecules by water+ carbon monoxide radiolysis: Relevance to comets, icy satellites, and interstellar ices. *Icarus* **140**, 451-461 (1999).

48. Burchill C, Ginns I. Radiation-induced oxidation of 2-propanol by hydrogen peroxide in aqueous solutions. *Canadian Journal of Chemistry* **48**, 1232-1238 (1970).

49. Chawla OP, Arthur N, Fessenden RW. Electron spin resonance study of the photolysis of aqueous sulfite solutions. *The Journal of Physical Chemistry* **77**, 772-776 (1973).

50. Fowles P. Pulse radiolytic induced transient electrical conductance in liquid solutions. Part 4.—The radiolysis of methanol, ethanol, 1-propanol and 2-propanol. *Transactions of the Faraday Society* **67**, 428-439 (1971).

51. Freeman GR. Radiation chemistry of ethanol: A review of data on yields, reaction rate parameters, and spectral properties of transients. (1974).

52. Hecquet MF, Roux JC, Simonoff GN, Sutton J. Radiolyse de l'isopropanol et des mélanges eau-isopropanol. *International Journal for Radiation Physics and Chemistry* **1**, 529-540 (1969).

53. Mason SA, Arey J, Atkinson R. Kinetics and Products of the OH Radical-Initiated Reaction of 1,4-Butanediol and Rate Constants for the Reactions of OH Radicals with 4-Hydroxybutanal and 3-Hydroxypropanal. *Environmental Science & Technology* **44**, 707-713 (2010).

54. Mezyk SP, Madden KP. Self-Recombination Rate Constants for 2-Propanol and tert-Butyl alcohol Radicals in Water. *The Journal of Physical Chemistry A* **103**, 235-242 (1999).

55. Ponomarev AV, Vlasov SI, Kholodkova EM. Effect of Boiling on the Radiolysis of 1-Propanol. *High Energy Chemistry* **53**, 314-320 (2019).

56. Russell JC, Freeman GR. Reactions of the primary reducing species in the radiolysis of liquid 2-propanol. *The Journal of Physical Chemistry* **72**, 808-815 (1968).

57. Sherman WV. The γ-Radiolysis of Liquid 2-Propanol. Effect of Nitrous Oxide and Sulfuric Acid. *The Journal of Physical Chemistry* **70**, 667-672 (1966).

58. Sweeney M. RADIATION CHEMISTRY OF ISOPROPYL COMPOUNDS (thesis).). California. Univ., Berkeley. Lawrence Radiation Lab. (1962).

59. Korpanty J, Gnanasekaran K, Venkatramani C, Zang N, Gianneschi NC. Organic solution-phase transmission electron microscopy of copolymer nanoassembly morphology and dynamics. *Cell Reports Physical Science*, 100772 (2022).

60. Rodgers M. Pulse radiolysis studies of acetone solutions. *Transactions of the Faraday Society* **67**, 1029-1040 (1971).

61. Woehl TJ, Abellan P. Defining the radiation chemistry during liquid cell electron microscopy to enable visualization of nanomaterial growth and degradation dynamics. *Journal of Microscopy* **265**, 135-147 (2017).

62. Korpanty J, Parent LR, Gianneschi NC. Enhancing and Mitigating Radiolytic Damage to Soft Matter in Aqueous Phase Liquid-Cell Transmission Electron Microscopy in the Presence of Gold Nanoparticle Sensitizers or Isopropanol Scavengers. *Nano Letters* **21**, 1141-1149 (2021).

63. Janik I, Ulanski P, Hildenbrand K, Rosiak JM, von Sonntag C. Hydroxyl-radical-induced reactions of poly(vinyl methyl ether): a pulse radiolysis, EPR and product study in deoxygenated and oxygenated aqueous solutions. *Journal of the Chemical Society, Perkin Transactions 2*, 2041-2048 (2000).

64. Kozicki M, Kujawa P, Rosiak JM. Pulse radiolysis study of diacrylate macromonomer in aqueous solution. *Radiation Physics and Chemistry* **65**, 133-139 (2002).

65. Ulanski P, Bothe E, Hildenbrand K, Rosiak JM, von Sonntag C. Hydroxyl-radical-induced reactions of poly(acrylic acid); a pulse radiolysis, EPR and product study. Part I. Deoxygenated aqueous solutions. *Journal of the Chemical Society, Perkin Transactions 2*, 13-22 (1996).

66. von Sonntag C. Free-radical-induced chain scission and cross-linking of polymers in aqueous solution—an overview. *Radiation Physics and Chemistry* **67**, 353-359 (2003).

67. Pedersen JS. Form factors of block copolymer micelles with spherical, ellipsoidal and cylindrical cores. *Journal of Applied Crystallography* **33**, 637-640 (2000).

68. Doucet M*, et al.* SasView version 5.0. 3. *Zenodo doi* **10**, (2020).

69. Menon S, Manohar C, Rao KS. A new interpretation of the sticky hard sphere model. *The Journal of chemical physics* **95**, 9186-9190 (1991).

70. Baxter R. Percus–Yevick equation for hard spheres with surface adhesion. *The Journal of chemical physics* **49**, 2770-2774 (1968).

71. Kotlarchyk M, Chen SH. Analysis of small angle neutron scattering spectra from polydisperse interacting colloids. *The Journal of chemical physics* **79**, 2461-2469 (1983).

72. Zhang S, Adamson DH, Prud'homme RK, Link AJ. Photocrosslinking the polystyrene core of block-copolymer nanoparticles. *Polymer Chemistry* **2**, 665-671 (2011).
