# Supplementary material for: Upper critical solution temperature polymer assemblies via variable temperature liquid phase transmission electron microscopy and liquid resonant soft X-ray scattering
Source: Nat Commun. 2023 Jun 10;14:3441. doi: 10.1038/s41467-023-38781-2 (PMC10257671; doi:10.1038/s41467-023-38781-2)
Supplement: Supplementary file 3 — Description of Additional Supplementary Files [file 41467_2023_38781_MOESM3_ESM.pdf]

**Description of Additional Supplementary Files**

File Name: Supplementary Movie 1
Description: LCTEM damage experiment on PEG-b-PEGMeA-co-PEGPhA-b-PS in IPA (1.42 $e^{-}$ $Å^{-2}$ $s^{-1}$, 15000 $e^{-}$ $Å^{-2}$)

File Name: Supplementary Movie 2
Description: LCTEM damage experiment on PEG-b-PEGMeA-co-PEGPhA in IPA (1.42 $e^{-}$ $Å^{-2}$ $s^{-1}$, 15000 $e^{-}$ $Å^{-2}$)

File Name: Supplementary Movie 3
Description: LCTEM damage experiment on PEGMeA in IPA (1.42 $e^{-}$ $Å^{-2}$ $s^{-1}$, 15000 $e^{-}$ $Å^{-2}$)

File Name: Supplementary Movie 4
Description: LCTEM damage experiment on PS in IPA (1.42 $e^{-}$ $Å^{-2}$ $s^{-1}$, 15000 $e^{-}$ $Å^{-2}$)

File Name: Supplementary Movie 5
Description: LCTEM damage experiment on PDEGEA in IPA (1.42 $e^{-}$ $Å^{-2}$ $s^{-1}$, 15000 $e^{-}$ $Å^{-2}$)
